# A Medical Causerie

**Published:** 1915-02-20

**Authors:** 


### A MEDICAL CAUSERIE.

Rumours have been prevalent for some little time that the annual meeting of the British Medical Association, arranged to be held at Cambridge in July, would not take place, and this has been officially confirmed. There will be much disappointment, but in the circumstances no other decision was possible. Once more it is the war which is responsible. Cambridge just now has no time or attention for the organisation of a series of scientific meetings, not to speak of the social functions which the occasion would demand. It is more like a military camp than a University city. Even professors and dons are learning to form fours and to stand at ease by numbers. The undergraduates are gone to the wars, or are getting ready to go there. There is much local pride in the arrangements for the wounded, and the military hospital is very much up to date. An invalided soldier, contemplating it as his future residence, was heard mournfully to exclaim, "More open-air treatment, I suppose."

Another disappointment is the intimation that the issue of Sir Clifford Allbutt's forthcoming book on "Arterial Disease and Angina Pectoris" is to be delayed. The volumes, for there are to be two of them, were expected in the autumn, and it is understood that they are actually in print. But it is now announced that there is to be further delay. Presumably the publishers know their own business best, but it is not easy to appreciate the reasons for their decision. A book by Sir Clifford Allbutt is always sure of an audience, and in this instance there is the attraction that the subject is one on which he speaks with very special knowledge and authority.

The past weeks have brought a pleasing prospect for the lovers of controversy by correspondence. Some few months ago there seemed every chance of a fierce encounter over the virtues of carbolic acid as a local dressing for wounds, but, though one of the protagonists openly invited someone to tread on the tail of his coat, the whole matter ended in smoke. Now the Army "rum ration" is the point of contest, and with two such experienced and notable combatants as Sir Victor Horsley and Dr. Mercier, some lively times have been expected. Indeed, the first battle has already been joined, and spectators have so far no cause to complain of the entertainment. The times are obviously propitious for the development of pugnacity, but it is to be hoped that evil example will not lead the combatants outside the rules of the game.

On the question of rum a curious observation is recorded by a Special Correspondent of the *Times*. Of course, the war has again the responsibility. Out of this has come an increased tax on beer; and the favourite beverage having consequently "gone up" in price, the prudent men of Kent will have none of it, and so have taken to drinking rum. The argument of the rural labourer is that beer has never been worth threepence a pint, and that it is not worth it now. Obviously, then, the sound economist will refuse an over-priced article. On the other hand, the twopence saved on the malt liquor is found by experience to be spent with advantage on rum. In Russia total abstinence seems to commend itself, but they know better than this in Kent. The result is one of those odd and unexpected consequences which so often, to the surprise of legislators, follow Acts of Parliament and official regulations.

Among the professional organisations suffering from the war must be numbered apparently the dignified institution known as the Royal College of Physicians of London. At the last "comitia" only three gentlemen were admitted to the Membership, so that presumably the number of candidates who entered for examination was unusually small.

The London post-graduation schemes, also, are feeling the strain badly. Indeed, two of them have frankly given up the struggle, though in one case the collapse was due to causes other than the present emergency. In some quarters great things are hoped for when the war is over, but not much can be expected until the whole question is tackled in a broad and liberal fashion and something like union of effort is secured.

A point of some importance to hospitals was raised at a recent meeting of the Medico-Legal Society. It arose in a discussion on a paper read by Sir John Collie, and dealing with the treatment of neurasthenia. The author recounted some successful experiences in dealing with neurasthenic patients—in receipt of a weekly allowance under the Workmen's Compensation Act—by gaining their admission to one of the hospitals for nervous diseases. One of the speakers who followed in the debate questioned whether public hospitals ought to be used for this purpose. He pointed out that the patients, so long as they were disabled, had a legal claim against their employers, which in most cases meant against an insurance company. It is therefore greatly to the interest of the insurance companies to get such persons quickly cured. But why should hospitals supported by voluntary subscription undertake gratuitous work for these companies? The point is obviously one of substance, and the interests concerned might well give it some attention.

Hospital Pharmacopœias and the British Pharmacopœia hardly stand on the same platform, but the new edition of the latter compels some revision of the non-official formulæ. In the composition of these there must needs be used some of the preparations which have been authoritatively modified in strength, and hence the quantitative value of the corresponding hospital prescription is changed. The medical staffs might give this matter their attention, for, unless they do so, the medicines they order will not be exactly the medicines they intend.

